# Intrusion detection using search-based learning optimized ensemble tree classifier model

**DOI:** 10.1371/journal.pone.0339822

**Published:** 2025-12-29

**Authors:** Afnan M. Alhassan, Nouf I. Altmami

**Affiliations:** Department of Computer Science, College of Computing and Information Technology, Shaqra University, Shaqra, Saudi Arabia; Nanjing Forestry University, CHINA

## Abstract

An Intrusion Detection System (IDS) is an important component of cybersecurity, meant to monitor malicious behaviour, detect, and respond to unauthorized activities in computer systems or networks. Generally, Intrusion detection (IDS) is classified into host-based IDS (HIDS) and network-based IDS (NIDS), which monitor individual devices and network traffic, respectively. Existing models faced certain limitations, including the dilemma of balancing false positives against false negatives, the challenge of adjusting to evolving threats, handling issues with high-dimensional information and encrypted traffic, and limited resource competence when dealing with privacy concerns. The proposed research work currently aims at developing an intrusion detection system that is more adaptive and effective to hinder these existing challenges and improve the security of digital environments. The study is related to applying an elaborate Search-based learning-optimized ensemble tree classifier (SBO-based ensemble tree classifier) for improving ID in Vehicular Ad Hoc Networks (VANETs). The ensemble classifier incorporates decision tree, random forest, extra tree, and eXtreme Gradient Boosting (XG Boost) classifiers, which are fused to provide a comprehensive interpretation of potential attacks within the VANET environment. Moreover, the research is enriched by incorporating Search-based learning optimization that takes advantage of their collective and adaptive nature. This innovative amalgamation attempts to perfect the aggregated response generated by the ensemble classifier, which fine-tunes the proposed model for effective intrusion detection. To facilitate the multi-dimensional orientation, four separate outputs, such as alpha, beta, gamma, and delta, were introduced, which allow the categorization of intrusion attacks based on specific types. More specifically, the experimental results illustrate that the proposed SBO-based ensemble tree classifier achieved superior performance with an accuracy of 96.56%, F1-score of 96.63%, FPR of 0.97, MCC of 0.97, Precision of 96.59%, Sensitivity of 96.68%, and Specificity of 96.52% for intrusion detection and outperforms the other existing methods using the BOT-IOT Dataset.

## 1. Introduction

Modern technology advancements have greatly improved communication across various fields, with Cyber-Physical Systems serving as a unique platform for sharing and transferring information through different channels [1]. This technology has led to significant progress in communication platforms, ultimately contributing to economic development. However, ensuring security and resilience remains a challenge and should be prioritized for enhancing security measures [2]. Communication failure occurs primarily due to security breaches and component malfunctions [3]. Due to the progress in communication, networking, and the Internet, different industries such as healthcare, transportation, social media, and industry now produce a vast amount of data daily. This data is classified as big data due to its significant size as well as its diverse range, rapid flow, and reliability [4]. Due to the immense volume of big data, identifying intrusions in such an environment becomes difficult due to the higher vulnerability present [5]. Denning et al. initially presented intrusion-detection systems in 1986, which identified anomalous behavior in networked systems [6]. The study and relevance of IDS continue to be important as big data evolves, networked systems grow faster, and intruders modify or innovate their techniques [7,8].

The convenience in businesses, organizations, and social communities is being aided by the rapid progress of computer networking technology [9]. Nevertheless, this development is also giving rise to a constant growth in internet security risks as vulnerabilities and attack methods multiply [10]. Therefore, it is important to implement security systems to prevent attacks and ensure the confidentiality, availability, and integrity of Internet communications. The most significant solution for network security is the use of IDS, which helps identify and restrict malicious network traffic [11]. There are two types of IDS, namely misuse-based IDS and anomaly-based IDS. Misuse-based IDS identifies attacks by comparing intrusion activity with preset patterns, which means it can only detect attacks that are already familiar [12,13]. On the other hand, anomaly-based IDS detects abnormalities in system behavior and can identify both known and unknown attacks [14]. The system has the ability to detect unfamiliar attacks by observing how they behave in a new network setting [15]. Additionally, this system is employed to safeguard the network against assailants. Furthermore, it identifies the flow of network traffic and categorizes it as either regular or irregular [16]. Anomaly detection involves creating a model of normal behavior and differentiating between normal and malicious patterns. In contrast to methods that detect signatures, this form of detection has the capability to identify attacks that are not previously known. Stateful protocol detection methods compare observed events to expected protocol behavior, enabling the identification of deviations [17,18].

Different machine learning techniques, such as neural networks, fuzzy logic, and support vector machines (SVMs) [19], have been explored for developing IDSs [20]. These techniques serve as classifiers to determine if incoming network traffic is normal or an attack. However, the rise in internet-based services has resulted in a significant surge in network traffic data, posing challenges for traditional tools in processing. Consequently, there is a need for a rapid and efficient ID system that can swiftly process large and intricate network data for effective ID [16]. It is worth noting that deep learning, which is a subset of Machine Learning (ML), has the potential to be applied to new problems that involve complex and high-dimensional data [21]. Additionally, deep learning algorithms facilitate the training of nonlinear models on extensive datasets, making them especially advantageous in the realm of network ID [22]. Deep learning is highly effective in processing and managing vast quantities of data, making it adaptable and proficient in diverse network settings. Ideally, it should possess the ability to effectively identify novel attack patterns and demonstrate competency in generalizing [23]. The convolutional models obtained certain limitations in terms of cyber-attacks, anomaly detection, false positives, high computation time, and overfitting issues [24]. To overcome these issues, this research developed a simple machine-learning model to improve intrusion detection in VANETs. The model integrates an advanced tree-based ensemble classifier with a hybrid optimization that supports the system and overcomes the problems of computation overheads and adaptability in intrusion detection. The main contributions of the intrusion detection system are explained below.

a) **Search-Based Learning Optimization:** The proposed machine learning model employed the hybrid Search-Based Learning optimization algorithm, which integrates Teaching-Learning Optimization (TLBO) and Crow Search Optimization (CSO) to improve the flexibility and contributive search tactics. The hybridization of nature-inspired algorithms tunes the model to achieve high accuracy while minimizing false errors. The benefits of the hybridized SBO algorithm make the intrusion detection model robust against falsely classified insider threats, a remarkable contribution towards securing vehicular communication systems.
b) **SBO-Based Ensemble Tree Classifier:** The proposed ensemble tree classifier employed four tree-based machine learning models, such as decision tree, random forest, extra tree, and XG Boost, which were iteratively tuned by varying the weights assigned to each classifier based on training for accurate intrusion detection. The SBO technique controls the parameters of the ensemble tree classifier to attain high detection accuracy for intrusion detection, while minimizing false positives and improving the convergence rate.

The manuscript follows a specific organizational structure, with Section 2 providing details about the Literature review, challenges, and current works. In Section 3, the methodology for an effective intrusion detection approach utilizing a tree-based ensemble classifier model is explained. Section 4 showcases the use of search-based learning optimization. Section 5 involves the results and analysis of the outcomes obtained from the experiment, while Section 6 provides the conclusion section.

## 2. Literature review

Muhammad Asif et al. [1] introduced an ML Technique for detecting intrusions in large networks. The technique leverages both MapReduce and ML techniques to efficiently handle large amounts of data and identify intrusions. The model demonstrated higher accuracy in processing large datasets and also generated numerous false alarms. A test bed platform created by K. V. V. N. L Sai Kiran et al. [2] to investigate and carry out Internet of Things (IoT) attacks on the network. The algorithms used in the platform demonstrated a high level of accuracy in classifying data. One of the main challenges in building an IDS using ML principles is the generation of a realistic and high-quality training dataset. It is crucial to have a continuous flow of data of good quality during the attack process for effective interception. In the research conducted by Mohammad Mehedi et al [3], a powerful integration of deep learning techniques was introduced for the identification of network intrusions. Their approach involved the use of a Convolutional Neural Network (CNN) to extract significant characteristics from the extensive data of IDS. Additionally, they utilized a hybrid model to maintain the long-term relationships among these characteristics, thereby preventing excessive emphasis on recurrent connections and overfitting. Additionally, the model had a very low execution time. It should be acknowledged that this model is prone to producing false alarms. The detection of intrusion information was addressed by Weijian Fang et al. [4] through the utilization of a machine learning technique. Their method incorporated the strengths of the Elman neural network and robust Support Vector Machine (SVM) noise data elimination to effectively mitigate security threats within information systems and safeguard their overall integrity. Through simulation experiments, they proved that the model had a minimal rate of false positives. However, it is vital to improve the speed of the ID system to meet the requirements of network communication. B. Riyaz and Sannasi Ganapathy [5] introduced a novel ID system for enhancing data communication security in wireless networks. Their model effectively identifies and detects intruders, achieving a high level of accuracy in predicting attacks. They addressed the issue of data dimensionality, improving the model’s performance. However, the model also has a high rate of false alarms. Mohammad Noor Injadat et al. [6] developed a model aimed at addressing malicious online behavior. Their model balances detection performance, accuracy, and time complexity while reducing computational complexity. Nevertheless, the presence of redundant or irrelevant features hinders the model training process and its detection capabilities. Wei Zong et al. [8] presented a technique for the representation of network ID information in a three-dimensional visualization, resulting in reduced computation costs and improved detection rates. However, this model also encountered significant issues with overfitting. Soosan Naderi Mighan and Mohsen Kahani [9] presented a novel method for cybersecurity ID. Their approach involved combining a stacked auto-encoder network for feature extraction with an SVM classifier. This combined technique offered improved flexibility and security, enabling a faster and more efficient detection system. Additionally, the execution time of their method was notably shorter compared to other approaches. Nonetheless, the large volume of traffic data posed a substantial challenge for ID. Zhen Dai et al. [25] presented a model to detect zero-day attacks from unseen data, integrating a trained autoencoder with XGBoost and Random Forest. The model effectively captured intrinsic features during training, which led to the robustness of the model in handling anomaly detection, and the model also struggled with parameter sensitivity and scalability issues. Abdelwahed Berguiga et al. [26] developed a deep learning model-based ID system for the Internet of Medical Things (IoMT). The model integrated a CNN for feature extraction and a Long Short-Term Memory (LSTM) for sequence data prediction, which effectively distinguished normal and anomalous traffic and minimized the false positives. However, the model is affected by latency issues in multiclass situations due to limited resources. Ahlem Harchay et al. [27] proposed a hybrid Deep Learning-Based IDS with the Routing Protocol for Low-Power and Lossy Networks. The model employed CNN with LSTM to handle evolving threats by focusing on low-level feature representations, periodic retraining, and anomaly detection. Although the model achieved high efficiency still struggled with computational complexity. H.M. Rai et al. [28] presented an AI-enhanced Conventional rule-based Network IDS, which utilized machine learning techniques for the classification of normal and abnormal threats in real time. This model has the best capability in threat detection and protection as advanced cyber threats arise all the time. However, the AI-NIDS model generated false positives or false negatives intrusions and required significant resources with high costs.

### 2.1 Challenges

- Nevertheless, it remains challenging to model Cyber-attacks, although modern security datasets may encompass various security features that may be of lesser importance or unnecessary [1].
- The primary hurdle in constructing IDS using machine learning principles lies in developing a credible and top-notch training dataset. It is imperative to ensure a steady flow of high-quality data in the network to intercept attacks effectively, as interception is only possible during uninterrupted data flow. Describing the user or normal system usage is the primary hurdle in anomaly detection. By doing so successfully, it becomes possible to enhance the accuracy of attack detection while minimizing false alarms [2].
- In order to keep up with the requirements of network communication, it is essential to improve the speed of the ID system. The emphasis should be on minimizing both false negatives and false positives for enhanced safety and accuracy [3].
- The system faces significant challenges concerning security, abnormality, and service failure. Thus, it is essential to develop an effective system that can overcome these challenges [4].

### 2.2 Motivation

Research motivation for intrusion detection comes from the constantly changing and expanding cyber threat landscape. As computer technology advances, the complexity and range of cyber-attacks on computer systems and networks also progress. Intrusion detection is the crux of digital security because it locates and responds to unauthorized access, data breaches, as well as any other form of espionage in digital environments. The rationale for continuous research is to stay ahead in the game of always-changing threats, which include new attack techniques and vulnerabilities. In addition, with the growing size and complexity of network infrastructure, there is a need for stronger, adaptive, & efficient ID systems. The exploration of advanced algorithms, ML techniques, and innovative methodologies aimed to enhance the accuracy, speed, and robustness of Information Technology (IT) refers to researchers’ motivations for securing increasingly connected digital assets. The outcome would be the creation of innovative technologies that are capable enough to effectively detecting and dealing with various cyber threats to ensure information systems maintain their integrity and confidentiality.

## 3. Methodology for proposed intrusion detection using search learning optimization-based ensemble tree classifier model

**Fig 1:**
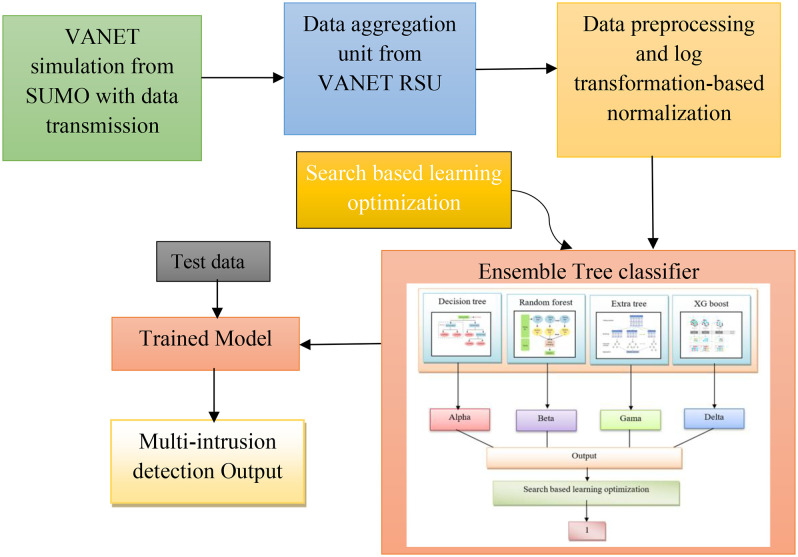
Architecture of the proposed SBO-Based Ensemble Tree Classifier model for intrusion detection.

The main purpose of this study is to design an effective intrusion detection model that would not only be able to detect possible attacks in VANETs but also distinguish them by type. The study starts with an exhaustive VANET simulation based on Simulation of Urban Mobility (SUMO), where information from vehicles is delivered to a data aggregation unit at the VANET Roadside Units (RSUs). Then the data goes through several preprocessing steps, such as normalization by log transformation. One of the main contributions of the intrusion detection system is a tree-based ensemble classifier that consists of a combination of Decision Trees, Random Forest, Extra Tree, and XG Boost, in which in total of 4 completely different outputs are marked by alpha, beta, gamma delta. In this case, these outputs are aggregated into one numerical value and then fed to a trained optimized model created by incorporating the SBO with an ensemble tree classifier. The outcome of this process can ultimately be utilized to identify the category of a multi-label intrusion attack or establish if it is not facing any issues. This methodology involves a comprehensive and systematic approach to intrusion detection in VANETs that includes simulation, data handling, classification, and optimization for increased security in vehicular communication network systems. The architecture of the proposed SBO-Based Ensemble Tree Classifier model for intrusion detection is presented in Fig 1.

### 3.1 VANET simulation using SUMO

The research begins by first simulating VANETs with SUMO (Simulation of Urban Mobility). This simulation is made to mimic real-life situations where cars are used for transportation within the city. SUMO helps model many features, such as the movement of vehicle traffic flow and communication between vehicles at RSUs. In the VANET simulation, each vehicle produces data attributed to its operational movement, location, as well as its interactions with what’s around it. This information may encompass the vehicle speed, direction, as well as possibly data concerning communication with neighboring vehicles and RSUs. For good implementation of the intrusion detection model, it is necessary to generate false and realistic data, which would help in training as well as evaluation.

### 3.2 Data transmission to data aggregation unit at RSUs

The data is then sent from these vehicles to the aggregation unit of data, which is located in VANET RSUs. RSUs function as vital infrastructure structures in the VANET environment. They serve as communication centers that gather and process data from surrounding vehicles. This transmission phase has the essential role of centralizing information and preparing it for further analysis. The real-world data parameters are extracted from VANET by using Roadside Units (RSU). This initial phase forms the basis for the research and creates a dynamic, realistic environment through VANET simulation.

#### 3.2.1 Network model.

This study investigates a vehicular network on a straight highway section consisting of two lanes. The vehicles enter the section randomly, adhering to a Poisson distribution with an average rate of λ, and maintain a steady speed throughout. The set n is employed to represent the group of M vehicles that pass through the road during the study period, specifically η={1,2,...M}, while κ denotes the set of N evenly distributed Roadside Units (RSUs) along the section, labeled as (κ={1,2,....N}). The coverage areas of these RSUs do not overlap, and together they cover the entire road section. It is worth noting that each RSU is solely powered by a renewable energy source. The specific type of energy collection, whether it’s solar, wind, piezoelectric, or something else, is not important for this research since the main focus is on managing energy. It is assumed that each RSU can gather energy, denoted as $s_{l}\left(t\right)$, at a specific time t and store it in a limited storage device, usually a battery, as long as the storage device is not already full. Therefore, the inequality $s_{l}\left(t\right)+d_{l}\left(t\right)<f_{l}$ holds, where $d_{t}\left(t\right)$ represents the battery’s energy level at a given time t, and $f_{l}$ denotes the battery’s capacity for RSUl.

#### 3.2.2 Communication model.

Here only consider one-way communication from the RSUs to vehicles in the communication model. To simplify the process, time is divided into time slots, and each vehicle is given a slot by the RSU for communication at a constant data rate E. The allocation of slots is not based on the vehicle’s position in the coverage area. Consider the available time slots denoted as L, with each slot having a duration ofL. Additionally, when a vehicle crosses a road segment, it sends a communication request to the nearest RSU, providing information about its speed and position. This allows the RSU to estimate the energy cost of communication based on the distance between itself and the vehicle. With this information, the RSU can optimize its energy consumption by selecting the most appropriate slot to serve the vehicle. In this model, a log distance path loss model is used to measure the energy cost $D_{l}\left(n,t\right)$ of communication between the RSU l and the vehicle n at timet.


$$D_{l}\left(n,t\right)=T_{t,u}\left(l,t\right).LR=\frac{T_{uy}\left(n,t\right)LR}{TL_{o}{\left[\frac{d_{o}}{d_{l}\left(n,t\right)}\right]}^{\gamma }}=\frac{M_{o}\left(2^{\frac{D}{H}-1}\right)LR}{TL_{o}{\left[\frac{d_{o}}{d_{l}\left(n,t\right)}\right]}^{\gamma }}$$ (1)

The transmit power of RSU l at a given time slot is represented by $T_{ty}\left(l,t\right)$, while the received power at the vehicle n is denoted as $T_{uy}\left(n,t\right)$. The reference distance is indicated by $d_{o}$, and $d_{l}\left(l,t\right)$ refers to the distance between the RSU l and vehicle n during the time slot t. The value of the path loss exponent is denoted by γ, the duration of the time slot is indicated as L, the channel’s bandwidth is symbolized as H, the noise power is represented by $M_{o}$, and $TL_{o}$ signifies the path loss at the reference distance. Renewable energy is widely regarded as abundant and eco-friendly, with no additional financial burden for its harvesting. However, the main challenge lies in the fact that its availability is unpredictable compared to the stable electric grid. Hence, it is vital to efficiently employ the energy harvesting process for long-term optimization of network performance. The key difficulty lies in striking a balance between energy expenditure and conservation. There are times when it may be more beneficial to consume the energy when it is readily available, while at other times, it should be stored to avoid energy shortages. Hence, the main goal of the model is to examine the effect of factors such as the amount of stored and harvested energy, as well as communication requests, on scheduling decisions. When addressing this problem, consider it a scheduling matter to identify the optimal time slots for RSUs to establish communication with vehicles. The primary objective is to maximize the number of communication requests that are effectively catered to, placing priority on this as the primary measure of performance. To streamline the situation, the operation is conducted under the assumption that the network can handle delays, every RSU solely relies on energy harvesting, and the vehicles possess an equal number of communication requests along with equal priority.

### 3.3 Data preprocessing and log transformation-based normalization

In the data preprocessing phase, the aggregated information from VANET simulation and RSUs goes through a sequence of operations to prepare it for further analysis and model training. Initially, the preprocessing involves deep cleaning, where noisy or irrelevant data is identified and dealt with, such as clearing duplicates and rectifying errors. Handling missing values is then performed to address the gaps in the dataset, a typical situation that arises when dealing with real-life datasets. Further, the preprocessed data is applied to the log transformation.

After this preprocessing, a mathematical operation called log transformation, applying the natural logarithm to every data point, is performed on them. Log transformation is a feature transformation technique utilized for handling skewed data into normally distributed data. This technique is applied to stabilize variability and symmetry in the data. Normalization will then be conducted to scale values of different features into a similar range or reduce dimensionality, ensuring uniformity and preventing certain characteristics from overly influencing machine learning algorithms. Following this, the data is prepared for analysis with categorical variables transformed, new features generated, and information aggregated to capture relevant patterns. Collectively, these merged pre-processing techniques log transformation and normalization, increase the quality of the dataset, making it good enough for effective training and optimization in performance when using machine learning models such as the intrusion detection model in this research setting.

### 3.4 Working of tree-based ensemble classifier in intrusion detection

**Fig 2:**
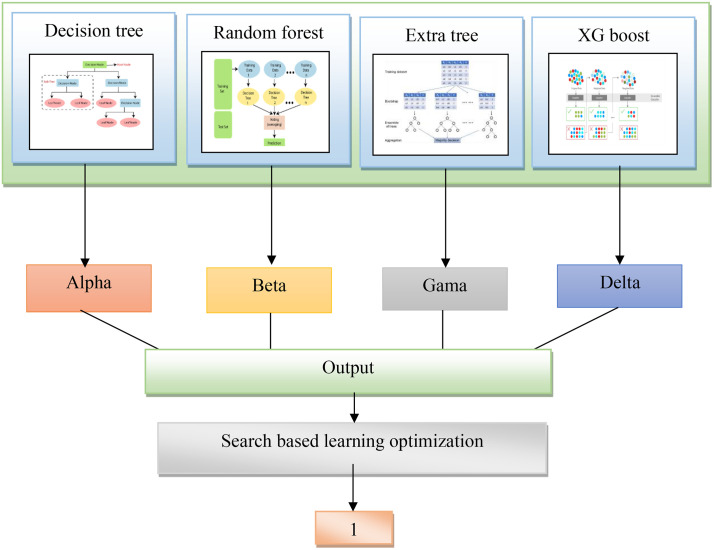
Architecture of the proposed ensemble tree- classifier.

The use of a tree-based ensemble classifier that includes decision trees, random forests, extra trees, and XG Boost algorithms presents multiple benefits in intrusion detection for VANET. Decision Trees are known for their interpretability and the ability to capture complex decision bounds. The incorporation of a random forest makes the model more resistant to overfitting by averaging out different trees trained on various split sets of data. An extra tree, in turn, is even more randomness-introducing at the stage of building up a tree and thus reduces variance and increases generalization. XG Boost is a gradient-boosting algorithm that sequentially enhances the accuracy of a model by compensating for errors that were made with previous trees. By merging these algorithms into an ensemble, the intrusion detection system gains from their collective strengths; this leads to a more robust, correct, and adaptive model. The ensemble approach is a powerful and well-suited solution for such complex tasks because these classifiers help to understand the intricate patterns associated with intrusion in VANETs. The ML classifiers utilized in the proposed model is comprehensively explained in following sections. The detailed architecture of the ensemble tree classifier is explained in Fig 2 as follows.

#### 3.4.1 Decision tree classifier.

A Decision tree (DT) is a straightforward classification algorithm that has the ability to handle nonlinear relationships between features and classes. The input data is split into two or more subsets based on specific criteria defined by the rules. This hierarchical structure facilitates decision-making as each subset becomes progressively more focused and discriminative. The splitting process continues iteratively until the subsets reach a desired level of granularity or meet specific conditions outlined by the rules. The input data is split into sub-nodes, which are then further split into more sub-nodes. This sequential binary subdivision eventually leads to the classification of the input data, with the target classes represented by the leaf nodes. However, there are some challenges with using DTs. One major issue is that they may not produce an optimal solution, as they rely on a single tree. Another common problem is overfitting, which needs to be considered when using DTs.

#### 3.4.2 Random forest classifier.

The Random Forest (RF) method is an approach that enhances the precision and effectiveness of decision trees by combining them. This is accomplished by integrating Breiman’s bagging sampling method with the technique of randomly selecting features. Bagging involves constructing decision trees in a group by randomly selecting samples from the training data, with replacements. These decision trees are used as fundamental estimators to determine the class label of an unlabeled instance through majority voting. Each base decision tree contributes a vote based on its predicted class label, and the instance is assigned the class label with the highest number of votes. RF is not affected by noise and overfitting issues.

#### 3.4.3 Extra tree classifier.

The Extra-Trees classifier follows the traditional top-down method in creating a group of decision trees, without pruning them. The extra-trees classifier employs a high degree of randomization when selecting attributes and cut points for splitting tree nodes. In some instances, the trees are entirely randomized, without consideration for the output values obtained from the training sample. This approach differs from other ensemble methods based on trees as it randomly chooses cut-points for node splitting and utilizes the entire training sample, rather than creating bootstrap replicas, to grow the trees. To determine the final prediction, the predictions of all the trees are merged and a majority vote is applied. The extra-trees classifier is based on the concept that the combination of complete randomization and ensemble averaging effectively reduces variance compared to methods that employ weaker randomization strategies. The utilization of authentic training samples, instead of creating duplicates through Bootstrap, helps in reducing bias as well. Additionally, this algorithm is well-known for its computational efficiency. The Extra Trees algorithm, similar to other algorithms, has been widely and diversely utilized in various studies.

#### 3.4.4 Extreme gradient boosting classifier.

XGBoost is an efficient and scalable implementation of the gradient tree-boosting algorithm. The main objective of the gradient boosting algorithm is to gradually enhance a classification model by incorporating weaker learners in order to minimize the loss function. By iteratively following the gradient and reducing errors from previous models, this algorithm constantly creates improved models. The XG Boost algorithm incorporates various components, including a model regularization technique to prevent overfitting, an algorithm for identifying splits that handle different types of data sparsity patterns, and a method for effectively handling weighted data known as the weighted quantile sketch algorithm. The text utilizes a column block structure to enable concurrent learning, a prefetching algorithm that is aware of the cache to collect and save gradient statistics, and computation blocks for handling extensive data.

The tree-based ensemble classifier produces four separate outputs, alpha, beta, gamma, and delta, which represent a multi-dimensional view of the output classification in the case of intrusion detection. Thus, each of such outputs probably reflects a certain feature or aspect concerning possible attack types and patterns within VANETs. This diversified output from decision trees, random forest, extra tree, and XGBoost implies that each classifier has different perspective values as well as strengths. The following step involves a careful merging of these outputs to form one numerical value via mathematical operation, usually addition. The purpose of this conglomeration is to integrate the distinct perceptions provided by each classifier into a unified and fully knowledgeable final result. After integrating the unique attributes of alpha, beta, gamma, and delta intrusion detection models can achieve a much more synoptic comprehension of complicated structures related to possible invasions in VANETs and improve their capacity to distinguish and identify different forms of safety threats efficiently.

An ensemble of classifiers is typically defined by a mathematical equation between the outputs that involves a weighted summation. The outputs for each output are multiplied by a weight, and then the sum of these weighted outputs produces an ensemble result. The weights are defined according to the performance or dependability of every single classifier.

Assuming alpha,beta,gamaanddelta represent the outputs of the decision trees, random forest, extra tree, and XGBoost, respectively, the combined output can be expressed mathematically as follows:


$$O=weight_{alpha}.alpha+weight_{beta}.beta+weight_{gama}.gama+weight_{delta}.delta$$ (2)

In this regard, $weight_{alpha},\;weight_{beta},weight_{gama},weight_{delta}$ weights are assigned to the outcomes of decision trees, random forest, extra tree, as well as XGBoost is the individual output from each of its respective classifiers.

The weights $weight_{alpha},\;weight_{beta},weight_{gama},weight_{delta}$ are usually determined through the model stacking or optimization process, where a validating set’s response to each classifier is taken into consideration. The weights can be set to emphasize the impact of better-performing classifiers in that particular problem setting.

## 4. Search-based learning optimization

The optimization phase requires the algorithm to iteratively tune weights assigned to each classifier based on how well an ensemble performs on a training set. The optimization technique is searching for a set of weights that maximizes predictive accuracy, minimizes error, or meets some other predefined goal. The merge of CSO and TLBO algorithms generates the advanced SBO algorithm with the characteristics of collaborative exploration and mutual knowledge, which helps in ID. Combining all these features, a crow optimization model that is informed by crow behavior would be able to use collective memory and cooperative search approach while borrowing the learning as well as interactive elements of TLBO. This combination may result in an optimization algorithm that not only taps into the wisdom of crowds but also adapts and learns from an evolving landscape, ultimately promoting a more intelligent or adaptable search for optimal solutions in complex problem spaces.

### 4.1 Inspiration

Crow attributes incorporated into optimization algorithms like CSO inspire intelligent and cooperative behavior from nature. Crows have individual problem-solving skills and adaptive strategies recognized in groups. This integration makes us think about the power of collective intelligence in situations where a problem must be solved. Watching how crows cooperate in distributing information towards a common cause can make us curious to investigate collaborative techniques when optimizing, leaving the impression of collective learning through successive improvement. In addition, TLBO is inspired by the things that happen in a classroom. The concept of learners increasing their standings through interactions with a teacher and among themselves can be paralleled in educational settings to collaborative learning environments. This gives us an impetus to leverage technical principles such as cooperation, knowledge sharing, and adaptability in algorithmic optimization. The idea of the teacher-student interaction concept prompts us to investigate how learning from both mentors and peers could improve problem-solving strategies, reflecting such variability and flexibility in natural learning processes. The mathematical formulation of the proposed SBO algorithm is explained as follows.

i) **Initialization:** Optimization algorithm is initialized by a random solution based on the weights and bias that depend on the solutions in all members of a population. To further improve this basic starting point, it is wise to incorporate diverse strategies that encourage diversity and exploration, as well as a wide addressing of the solution space.

$$X^{t}=l+X_{rand}\left(u-l\right)$$ (3)

Here, u and l denotes the upper and the lower bands of the solution, $X_{rand}$ denotes the random position.

ii) **Fitness evaluation:** While taking the crow characteristics in the TLBO algorithm, the fitness function is evaluated to improve the convergence rate. Here, the fitness function accuracy is maximized to achieve accurate intrusion detection.

$$Fit\left(X^{t}\right)=\max\left(Accuracy\left({X_{i}}^{t+1}\right)\right)$$

Where, $X^{t}$ denotes the initial solution and ${X_{i}}^{t+1}$ denotes the optimal solution

iii) **Exploration phase:** if $≥0.5, $ denotes the tradeoff factor.

In this phase, the learners become knowledgeable from the teacher, and in this way, their positions are better than other optimal solutions. Learners also engage in this dynamic of readjusting and improving their locations altogether by using the directives imparted to them by the teacher, thus helping refine and elevate optimization as a whole.


$$X_{i}^{t+1}=X_{i}^{t}+x_{1}\left(\alpha_{p}-TF.X_{mean}\right)$$ (4)

$$Where,\;r_{1}=\frac{\left|F\left(\alpha_{p}-F\left(X_{mean}\right)\right)\right|}{\left|F\left(\alpha_{p}\right)+F\left(X_{mean}\right)\right|}\;\;\varepsilon \left(0,1\right)$$ (5)

Here, TF denotes the teaching factor → round [1+rand(0,1)∈(0,2\rightleft{2−1}]

iv) **Exploitation phase:** if $<0.5

In this phase, the strengthening of learner solutions happens through connecting with each other, which implies a cooperative learning process where members within the learner population share information, strategies, and insights with one another. Through reciprocal interactions, every learner gains from the collective wisdom of the cluster, nurturing a dynamic and supportive relationship. The collaborative learning approach permits solutions to evolve and adapt collectively by taking advantage of the experiences that are varied as well and perspectives amassed inside the population. Knowledge of the learners is iteratively exchanged among them, optimizing the entire solution set, thus making it more adaptive and effective.

V) **Genuineness phase.**
  Subcase i) $\;if\;fit\left(X^{t}\right)\geq fit\left(Av_{X}\right)$

$$AV_{x}=\frac{\sum_{i=1}^{N}X_{i}^{t}}{\left[N\right]}$$ (6)

When the fitness of some learners is either equal to or equal than the mean of the population’s fitness, superior knowing beings initiate an influence process to help the rest learn how they could reach greater places. In this case, fitter individuals become teachers to pass their knowledge along to the rest of the learners for they all together improve the whole population’s fitness mark. This knowledge transfer mechanism encourages learning in a collaborative environment where the objective is merely raising all learners to improved positions and thus ensuring that the solution set evolves dynamically but efficiently.


$$X_{i}^{t+1}=X_{i}^{t}+r_{2}\left(X^{t}-AV_{x}\right)$$ (7)

Subcase ii) $if\;fit\left(X^{t}\right)<fit\left(AV_{x}\right)$

The solution uses its memorizing ability to subtly obtain the locations of other solutions that have limited energy and time. In other words, the learner uses their memorized knowledge in a strategic way to steal advantageous positions from less effective alternatives. This process, based on optimal memory usage, allows the learner to quickly adapt and enhance their status in the population while optimizing frames that work with minimal energy expenditure and time allocation.

Then,


$$X_{i}^{t+1}=\frac{\text{1}}{\text{2}}\left[X_{i}^{t}+r_{2}\left(AV_{x}-X^{t}\right)\right]+\frac{\text{1}}{\text{2}}\left[X_{i}^{t}+r_{1}\left(mem_{i}-X_{i}^{t}\right)\right]$$ (8)

Where, $mem_{i}$ denotes the memory matrix of $i^{th}$ solution


$$X_{i}^{t+1}=\frac{\text{1}}{\text{2}}\left[X_{i}^{t}+r_{2}\left(AV_{x}-X_{i}^{t}\right)+X_{i}^{t}+r_{1}\left(mem_{i}-X_{i}^{t}\right)\right]$$ (9)

$$X_{i}^{t+1}=\left[2X_{i}^{t}-r_{2}X_{i}^{t}-r_{1}X_{i}^{t}+r_{2}AV_{x}+r_{1}mem_{i}\right]$$ (10)

$$X_{i}^{t+!}=\frac{\text{1}}{\text{2}}\left[X_{i}^{t}\left(2-r_{1}-r_{2}\right)+r_{1}mem_{i}+r_{2}AV_{x}\right]$$ (11)

The given equation indicates that a learner tries to improve their position by smartly utilizing the available tool, such as using a memory matrix. This strategic use of intelligence involves tricking other solutions; herein lies the role that one’s memory plays. Through a cunning use of its stored knowledge, the learner strategically influences other solutions in terms of their positions. Such smart maneuvering effectively allows the learner to maximize its position, exhibiting a highly refined adaptation strategy founded on built-in cognitive strengths encoded into a memory matrix.

vi) **Termination condition:**

**Fig 3:**
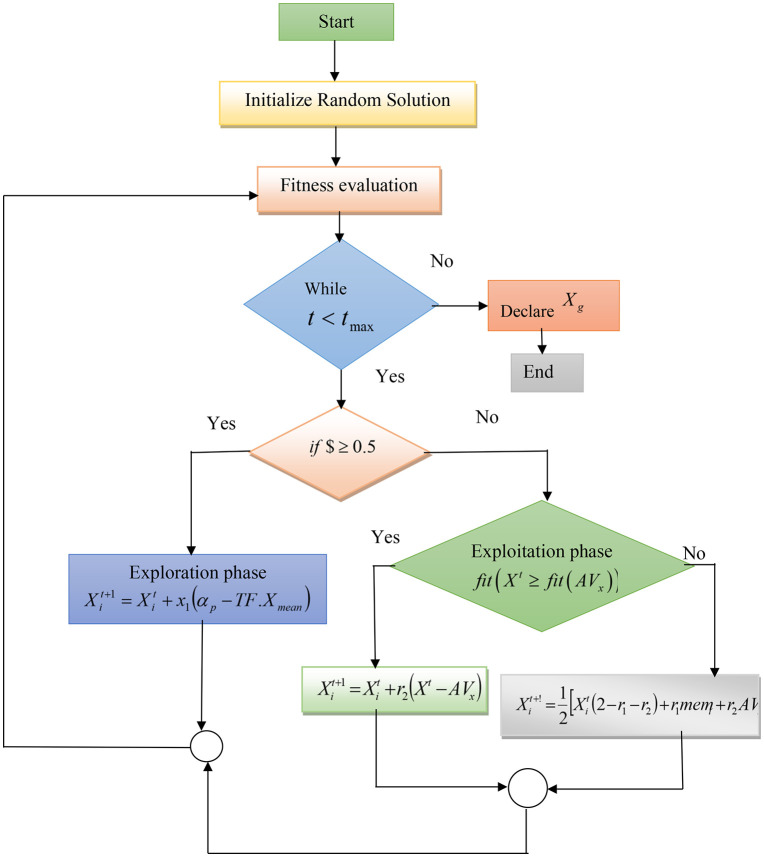
Flow chart for the proposed search-based learning optimization.

Following the evaluation process, the condition of termination $t<t_{\max}$ is evaluated to end the process that determines the best solution. When the optimal solution is obtained, the iterative process is terminated. Fig 3 illustrates the flow chart of the SBO algorithm utilized for intrusion detection.

## 5. Result and discussion

The intrusion detection model implements the SBO-based ensemble tree classifier and evaluates its effectiveness in comparison to other methods currently in use.

### 5.1 Experimental setup

The experiment on intrusion detection based on an SBO-based ensemble tree classifier is implemented on Windows 11 OS, utilizing 8GB RAM along with Python software. The initial parameters of the ensemble tree classifier includes the learning rate of 0.001, batch size of 128, number of epochs 100, activation function ‘ReLU’, regularization 12, dropout rate 0.5, the best threshold as splitter ‘best’, minimum samples split is 2, minimum samples leaf is 1, minimum weight fraction leaf is 0, maximum leaf nodes ‘none’, minimum impurity decrease to 0, ccp_alpha is 0, and criterion of Gini.

### 5.2 Dataset description

BOT-IOT Dataset [29]: There are two types of traffic in the environment, namely normal and botnet. The dataset is accessible in different formats, such as PCAP, Argus, and CSV files. To facilitate labeling, the files have been sorted and divided based on the type and subcategory of attacks. To make it easier to work with the dataset, a sample of 5% is extracted using select MySQL queries.
CIC-IDS-2017 Dataset [30]: The CICIDS2017 dataset consists of both harmless data and the latest commonly occurring attacks, mirroring real-world PCAP data. The dataset contains data from network traffic analysis carried out using CICFlowMeter. The classified flows in the dataset are organized according to different characteristics, including time stamps, source and destination IPs, source and destination ports, protocols, and the type of attack.

### 5.3 Performance metrics

The proposed BSO-based Ensemble tree classifier is evaluated to improve the performance using performance metrics such as accuracy, precision, F1-score, sensitivity, specificity, TPR, and MCC, which are explained below.

**Accuracy:** Accuracy is defined as the ratio of correctly classified attacks to total classified attacks, which is calculated as follows

$$Acc=\frac{TP+TN}{TP+TN+FP+FN}$$ (12)

**Precision**: Precision defines the ratio of correctly classified positive attacks to total classified positive attacks, which is mathematically represented as

$$\mathrm{Pr}e=\frac{TP}{TP+FP}$$ (13)

**F1-Score:** Defined as the harmonic mean of precision and recall value, balancing imbalanced data for intrusion detection, mathematically represented as

$$F1=\frac{TP}{TP+\frac{\text{1}}{\text{2}}\left(FP+FN\right)}$$ (14)

**Sensitivity:** Sensitivity is defined as the ratio of correctly classified positive attacks to total classified positive attacks, which is expressed as follows

$$Sen=\frac{TP}{TP+FN}$$ (15)

**Specificity:** Specificity is defined as a ratio of correctly classified negative attacks to total classified negative attacks, which is mathematically expressed as follows

$$Spe=\frac{TN}{TN+FP}$$ (16)

**FPR:** False Positive Rate is defined as the ratio of incorrectly classified positive attacks to total classified negative attacks, which is mathematically represented as

$$FPR=\frac{FP}{FP+TN}$$ (17)

**Matthews Correlation Coefficient (MCC):** MCC is a balanced measure for binary classifications to improve the imbalance class distribution, which is calculated using the formula.

$$MCC=\frac{\left(TP\times TN\right)-\left(FP\times FN\right)}{\sqrt{\left(TP\times FP\right)}\left(TP\times FN\right)\left(TN\times FP\right)\left(TN\times FN\right)}$$ (18)

Where, True Positive (TP) denotes the number of intrusions correctly identified, False Positive (FP) represents the number of false intrusions, True Negative (TN) denotes the number of negative intrusions that are correctly identified, and False Negative (FN) denotes the number of positive intrusions that are underreported.

### 5.4 Performance analysis

The proposed hybrid tree-based ensembled classifier is evaluated with existing tree classifiers based on the training percentage of 40%, 50%, 60%, 70%, 80%, and 90%. The proposed SBO-based ensemble tree classifier achieved high performance with TP-90% with performance metrics such as accuracy, precision, F1-score, sensitivity, specificity, TPR, and MCC explained in the following section.

#### 5.4.1 Performance analysis for intrusion detection using the BOT-IOT dataset.

**Fig 4:**
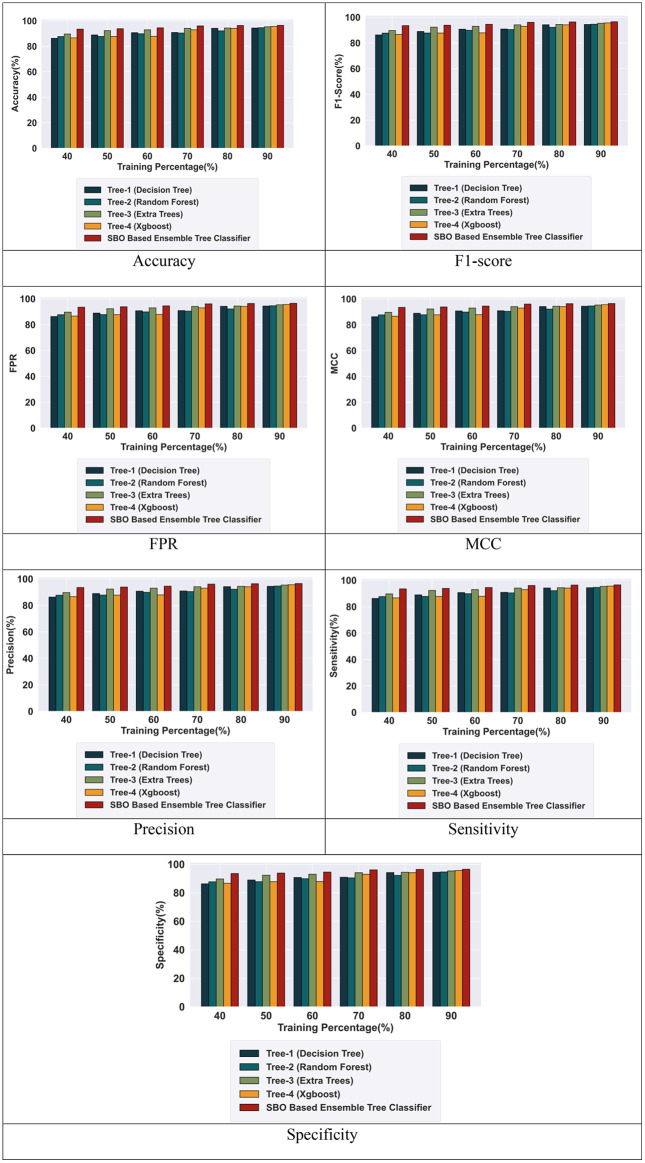
Performance analysis with the BOT-IOT Dataset.

**Fig 5:**
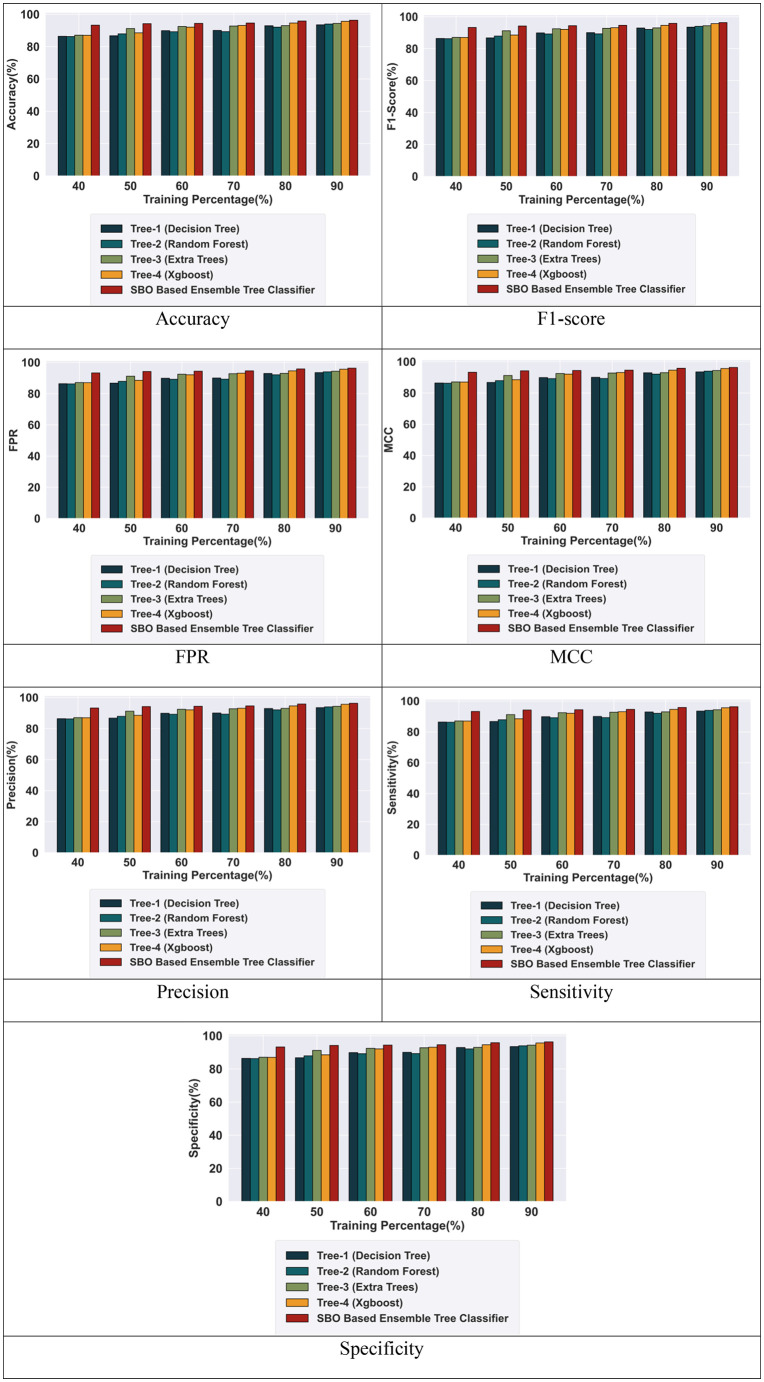
Performance analysis with the CIC-IDS-2017 Dataset.

Fig 4 illustrates the performance analysis of the proposed BSO-based Ensemble tree classifier based on Training percentage. The proposed model achieved high performance with 90% training for intrusion detection. Comparing Tree 1 (decision tree), Tree 2 (random forest), Tree 3 (extra tree), Tree 4 (XGBoost), the combined SBO-based ensemble tree classifier model achieves an accuracy of 94.46%, 94.70%, 95.42%, 95.66%, and 96.56%. Similarly, the model achieved F1-score of 94.40%, 94.60%, 95.39%, 95.69%, and 96.63%. The proposed model obtained FPR values of 0.945%, 0.947%, 0.954%, 0.956%, 0.956%. Likewise, the model achieved MCC values of 0.945%, 0.947%, 0.954%, 0.956%, and 0.966%. Additionally, the results display the precision results for the SBO-based ensemble tree classifier reaching 94.47%, 94.70%, 95.38%, 95.62%, and 96.59%. Also yielding a sensitivity of 94.33%, 94.50%, 95.40%, 95.76%, and 96.68%, all with a TP of 90. Finally, the specificity achieved by the SBO-based ensemble tree classifier is 94.63%, 94.89%, 95.32%, 95.44%, and 96.52%, respectively (Fig 5).

#### 5.4.2 Performance analysis for intrusion detection using CIC-IDS-2017 dataset.

### 5.5 Comparative methods

To demonstrate the effectiveness of the SBO-based ensemble tree classifier, a comparative examination is carried out utilizing various techniques including Support Vector Machine [31], Naive Bayes [32], Cat Boost Classifier [33], AdaBoost Classifier [34], Multi-Layer Perceptron [35], Fruit Fly Optimization Algorithm based CNN (FOA-CNN) [36], Recurrent Neural Network (RNN) classifier [37], and Multi-Blocks of CNN [38].

#### 5.5.1 Comparative analysis based on TP using BOT-IOT dataset.

**Fig 6:**
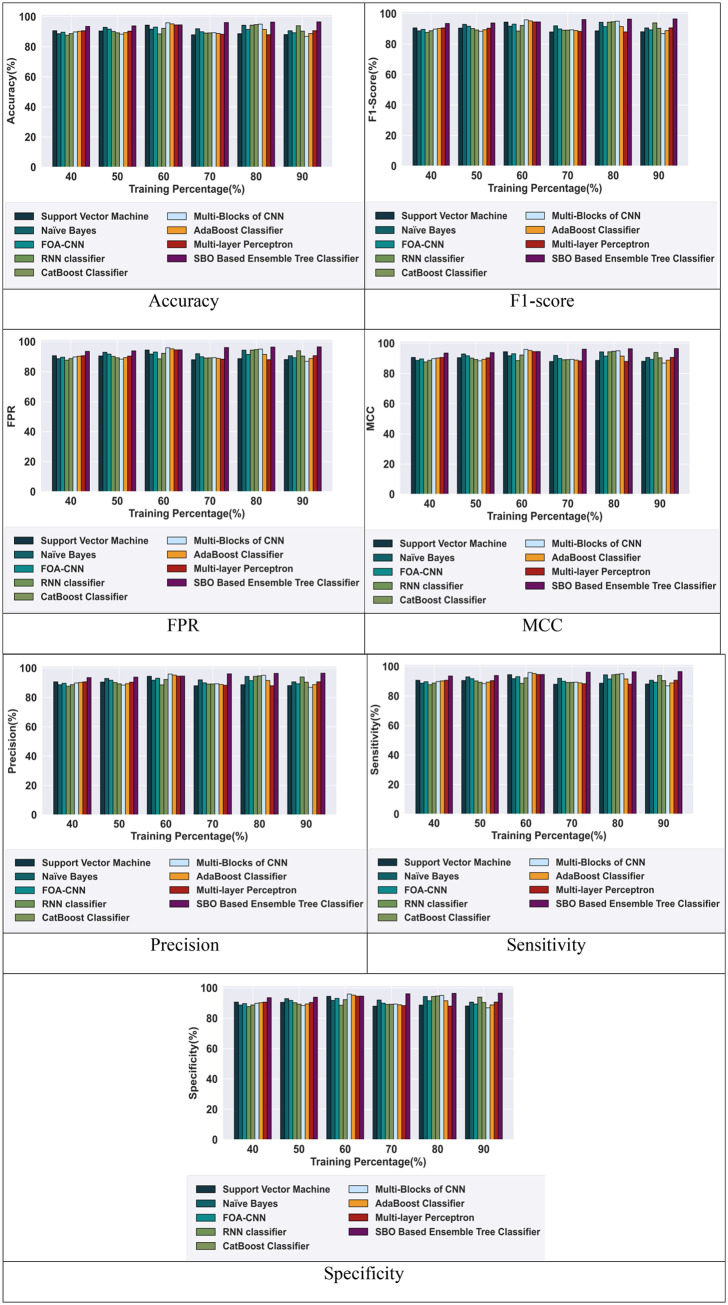
Comparative analysis with the BOT-IOT Dataset.

Fig 6 depicts the comparative analysis of the proposed SBO-based ensemble tree classifier. The proposed model achieved high metrics value and outperforms the other existing models based on 90% TP using the BOT-IOT Dataset, which enhances the performance of the model. The SBO-based ensemble tree classifier achieved a high accuracy of 96.56% outperforming the RNN classifier with an improvement of 2.71%. The model gained an F1-score of 96.63%, which is 2.84% higher than the RNN classifier. Similarly, the proposed model gained an FPR of 0.97, which is 2.64% higher than the RNN classifier. Likewise, the model attained a high MCC value of 0.97, which is 2.72% more than the RNN classifier. Additionally, the precision of the ensemble tree classifier is 96.59%, which outperforms the RNN classifier by 2.74%. The sensitivity of the ensemble tree classifier is 96.68%, which outperforms the RNN classifier by 2.94%. Also, the specificity of the ensemble tree classifier in intrusion detection is 96.52%, which outperforms the RNN classifier by 2.57%. However, the proposed SBO-based ensemble tree classifier achieved high efficiency for intrusion detection compared with other existing models.

#### 5.5.2 Comparative analysis based on TP using CIC-IDS-2017 dataset.

**Fig 7:**
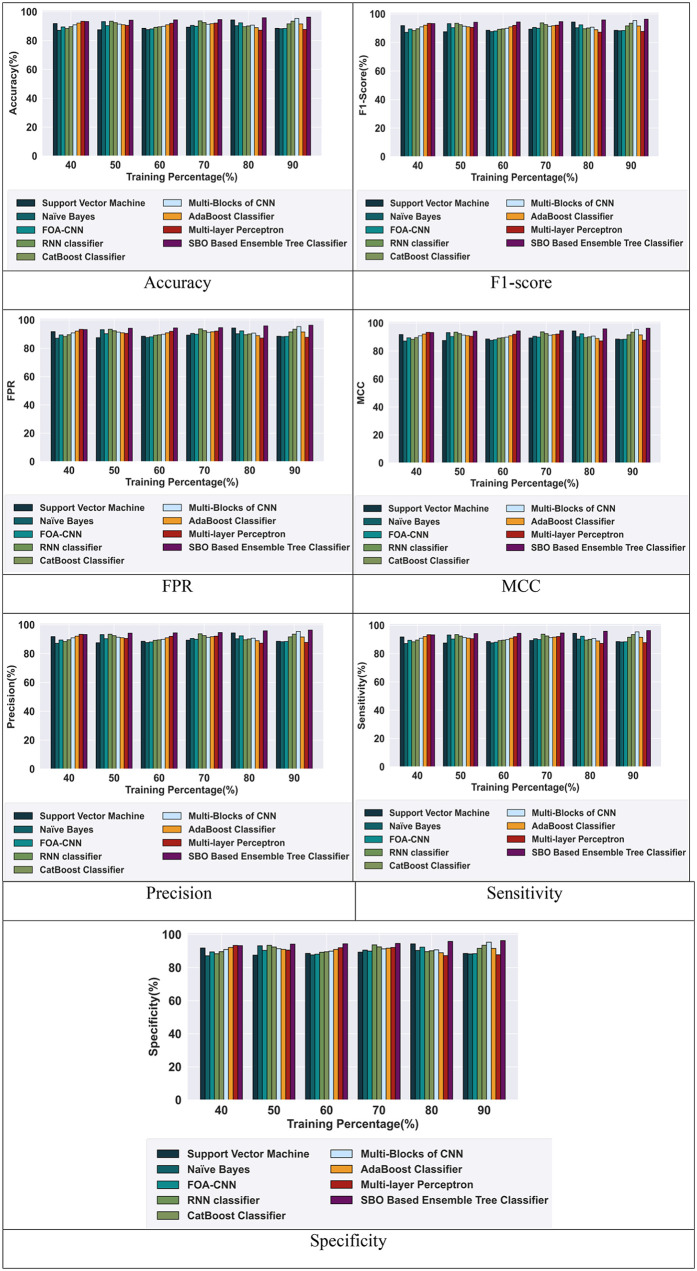
Comparative analysis with the CIC-IDS-2017 Dataset.

Fig 7 depicts the comparative analysis of the proposed SBO-based ensemble tree classifier. The proposed model achieved high metrics value and outperformed the other existing models based on 90% TP using the CIC-IDS-2017 Dataset, which enhances the performance of the model. The SBO-based ensemble tree classifier achieved a high accuracy of 96.33%, which outperforms the RNN classifier with an improvement of 4.87%. The model gained an F1-score of 96.45%, which is 5% higher than the RNN classifier. Similarly, the proposed model gained an FPR of 0.96, which is 4.74% higher than the RNN classifier. Likewise, the model attained a high MCC value of 0.96, which is 4.87% more than the RNN classifier. Additionally, the precision of the SBO-based ensemble tree classifier is 96.33%, which outperforms the RNN classifier by 4.87%. The sensitivity of the SBO-based ensemble tree classifier is 96.57%, which outperforms the RNN classifier by 5.13%. Also, the specificity of the SBO-based ensemble tree classifier in intrusion detection is 96.09%, which outperforms the RNN classifier by 4.61%. However, the proposed SBO-based ensemble tree classifier achieved high efficiency for intrusion detection compared with other existing models.

### 5.6 Comparative discussion

The proposed SBO -Ensemble Tree Classifier is evaluated with other existing models to enhance the efficiency for intrusion detection based on training percentage. In this specific situation, the effectiveness of the proposed SBO-based ensemble tree classifier is proven to be better than the existing models. More specifically, the experimental results show that the performance of the SBO-based ensemble tree classifier achieved remarkable outcomes with the BOT-IOT Dataset and CIC-IDS-2017 Dataset. Moreover, the model achieved high-performance values with an accuracy of 96.56%, F1-score of 96.63%, FPR of 0.97, MCC of 0.97, Precision of 96.59%, Sensitivity of 96.68%, and Specificity of 96.52% with the BOT-IOT Dataset. The conventional models used for intrusion detection have limitations such as poor anomaly detection, high false positive rate, high computation time, overfitting issues, computational overhead, and struggle with cyber-attacks. To overcome these challenges, the proposed machine learning model is designed for intrusion detection in VANET. The proposed model utilized advanced preprocessing techniques and an ensemble classifier, which effectively identifies intricate features that help to detect the intrusion and reduce overfitting issues. The integration of multiple classifiers with a hybrid BSO algorithm extract and learn intrinsic features; this simple, lightweight architecture reduces the computational overheads in the model. The evaluation results depicted that the proposed model achieved minimum computation time and high efficiency compared with other existing models, which is illustrated in Table 1.

**Table 1:** Comparative discussion of the SBO-Based Ensemble Tree Classifier.

Models		SVM	Naive Bayes	FOA-CNN	RNN classifier	Cat Boost Classifier	Multi-Blocks of CNN	AdaBoost Classifier	Multi-layer Perceptron	SBO-Based Ensemble Tree Classifier
**BOT-IOT Dataset**	Accuracy (%)	88.10	90.66	89.38	93.94	90.42	86.91	88.80	90.70	**96.56**
	F1-score (%)	88.11	90.57	89.34	93.89	90.57	87.25	88.92	90.59	**96.63**
	FPR	0.88	0.91	0.89	0.94	0.90	0.87	0.89	0.91	**0.97**
	MCC	0.88	0.91	0.89	0.94	0.90	0.87	0.89	0.91	**0.97**
	Precision (%)	88.10	90.66	89.38	93.94	90.42	86.91	88.80	90.70	**96.59**
	Sensitivity (%)	88.12	90.48	89.30	93.84	90.72	87.60	89.04	90.48	**96.68**
	Specificity (%)	88.08	90.84	89.46	94.04	90.12	86.21	88.56	90.92	**96.52**
**CIC-IDS-2017 Dataset**	Accuracy (%)	88.58	88.26	88.42	91.64	93.50	95.37	91.58	87.78	**96.33**
	F1-score (%)	88.78	88.46	88.62	91.63	93.46	95.29	91.54	87.78	**96.45**
	FPR	0.88	0.88	0.88	0.92	0.94	0.95	0.92	0.88	**0.96**
	MCC	0.89	0.88	0.88	0.92	0.94	0.95	0.92	0.88	**0.96**
	Precision (%)	88.58	88.26	88.42	91.64	93.50	95.37	91.58	87.78	**96.33**
	Sensitivity (%)	88.98	88.66	88.82	91.62	93.41	95.21	91.50	87.78	**96.57**
	Specificity (%)	88.18	87.86	88.02	91.66	93.59	95.53	91.66	87.78	**96.09**

### 5.7 Ablation study

**Fig 8:**
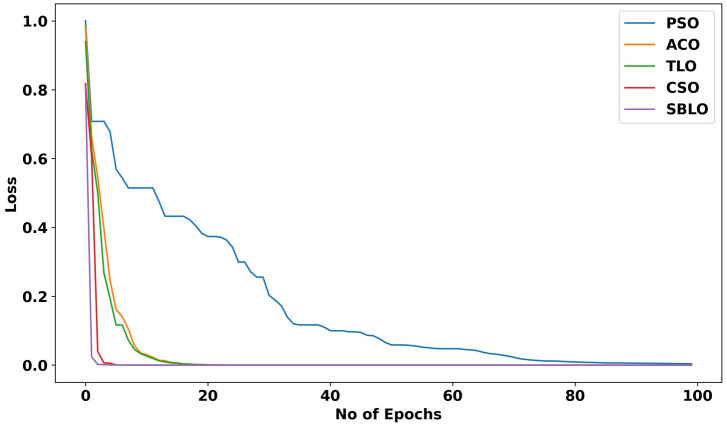
Ablation study of the Search-Based Learning Optimization.

The ablation study is conducted to evaluate the proposed Search-Based Learning Optimization with other existing algorithms based on the loss function at 100 epochs. The proposed algorithm integrated the Teaching-Learning Optimization (TLBO) and Crow Search Optimization. To evaluate the performance of the classifier, the proposed SBLO algorithm is compared with existing algorithms such as Particle Swarm Optimization (PSO) and Ant Colony Optimization (ACO). Moreover, the integration of the proposed SBLO optimization fine-tunes the ensemble tree classifier for achieving optimal solutions with minimum loss, which enhances faster convergence. The proposed SBLO algorithm is evaluated in multiple iterations to reach high efficiency. Fig 8 shows that the SBLO achieved a minimum loss of 0 at the 98^th^ epoch, which shows the effective performance of the optimization. Fig 8 illustrates the comparison of the SBLO algorithm with other existing optimization algorithms.

### 5.8 Computational complexity

**Fig 9:**
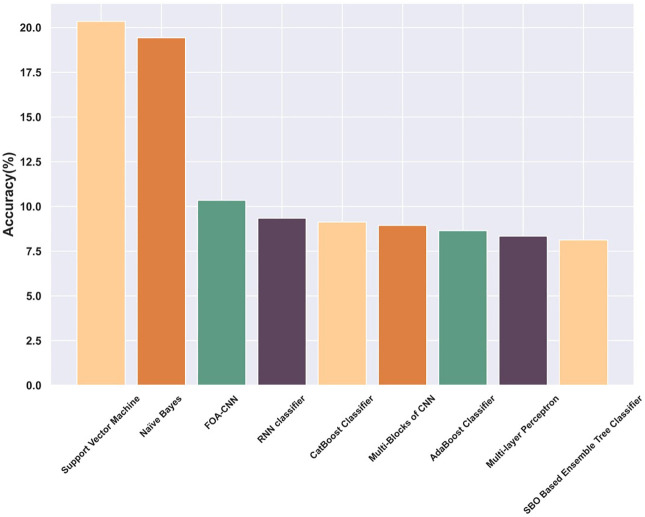
Computational Complexity.

The computational time complexity of the proposed SBO-based Ensemble Tree Classifier is estimated with other existing models based on the weights of the hyperparameters present in the Ensemble Tree Classifier that are tuned by the proposed SBO algorithm for 100 epochs. The optimization algorithm helps to attain the optimal solution with multiple iterations and achieves minimum computational time leading to a high convergence rate. The proposed model attained a less computational time of 8.12s, which enhances the efficiency of intrusion detection. Comparatively, other existing models gained high computation time, such as Support Vector Machine is 20.34s, Naive Bayes is 19.43s, FOA-CNN is 10.34s, RNN classifier is 9.34s, Cat Boost Classifier is 9.13s, Multi-Blocks of CNN is 8.94s, AdaBoost Classifier is 8.65s, and Multi-layer Perceptron is 8.34s. The computational complexity analysis is visually represented in Fig 9.

### 5.9 Feature importance analysis

**Fig 10:**
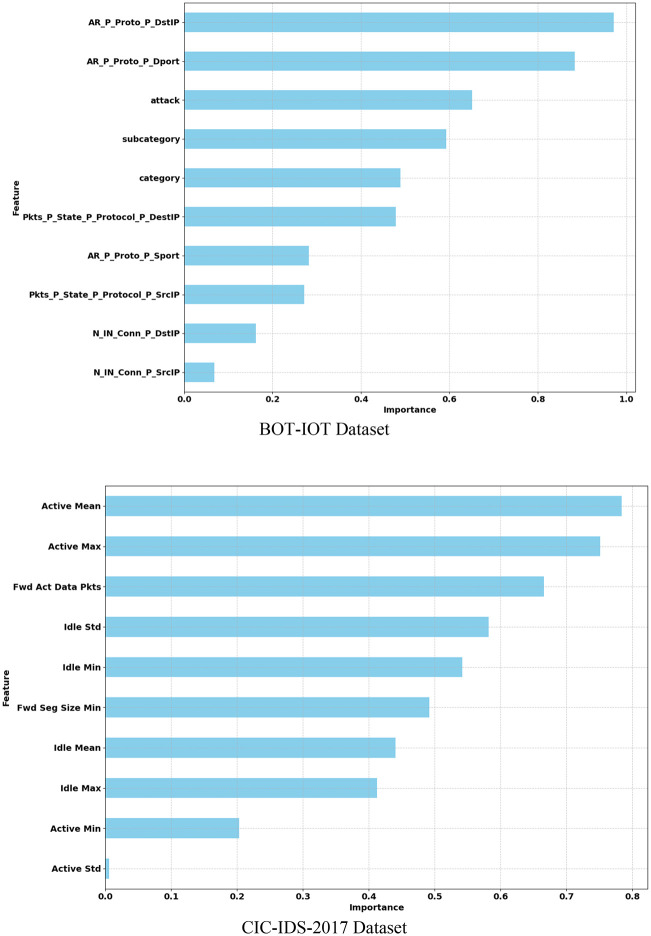
Feature Importance Analysis with SHAP.

The feature importance analysis utilizes the Shapley Additive explanations (SHAP) method to interpret machine learning models and analyze the contribution of significant features that influence accurate intrusion detection. Here, the feature importance analysis of the proposed SBO-Based Ensemble Tree Classifier is analyzed based on SHAP value using the BOT-IOT Dataset and the CIC-IDS-2017 Dataset. However, the BOT-IOT Dataset scored a high SHAP value of 0.97 for ‘AR_P_Proto_P_DstIP’ features, and the lowest value of 0.07 for ‘N_IN_Conn_P_SrcIP’ features. Similarly, the CIC-IDS-2017 dataset achieved a high SHAP value of 0.78 for active mean features, 0.76 with active max features, and the lowest value of 0.1 with active standard features. More specifically, the most important features that help to detect the intrusion are analyzed based on the SHAP value, which is illustrated in Fig 10.

### 5.10 Security analysis

**Fig 11:**
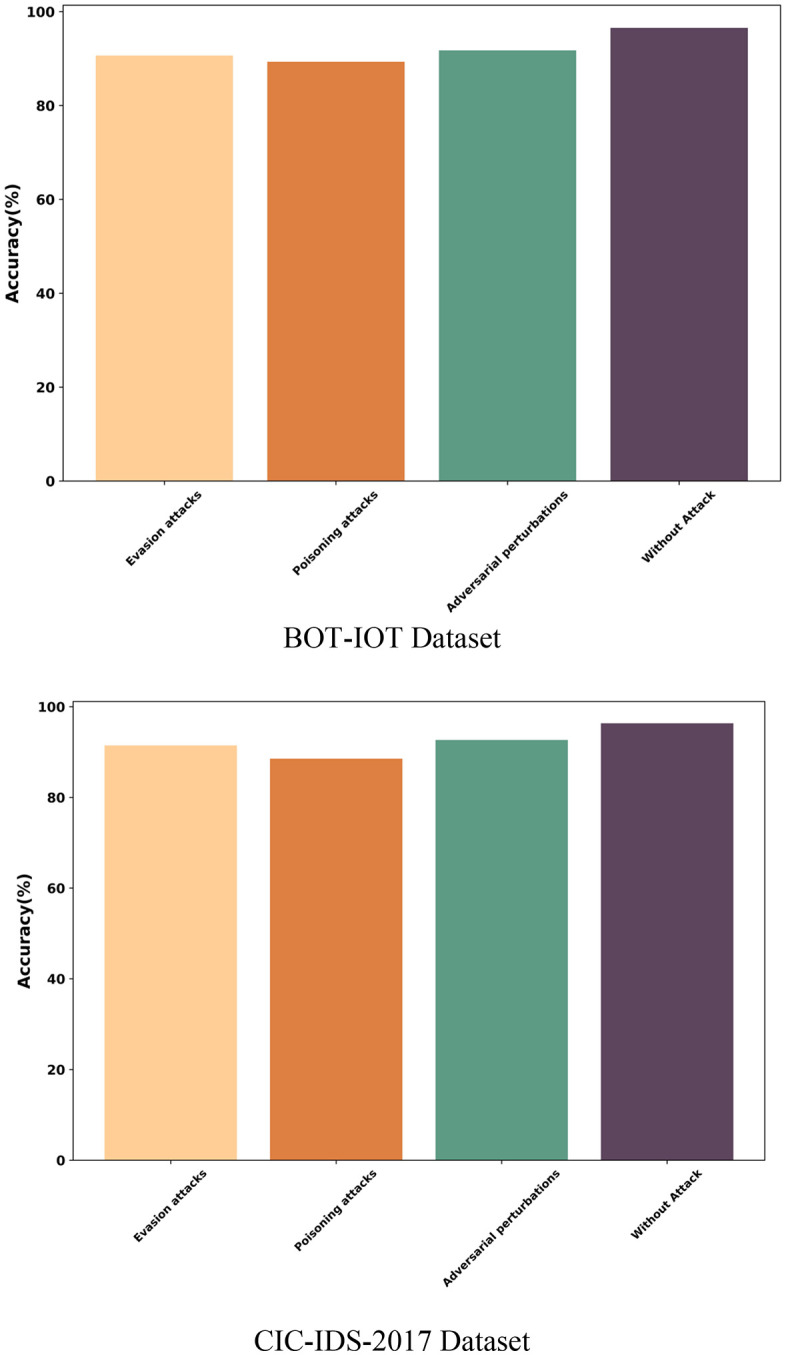
Computational Complexity.

The security analysis is conducted to evaluate risks associated with the security of the proposed SBO SBO-based ensemble Tree Classifier for intrusion detection with the Bot-IoT Dataset and CIC-IDS2017 Dataset by analyzing the various attacks. The Dataset is conducted with attacks such as Evasion attacks, Poisoning attacks, Adversarial perturbations, and Without Attack and attains the accuracy of 90.65%, 89.34%, 91.75%, and 96.56%, respectively. In the process of intrusion detection, the CIC-IDS2017 Dataset is evaluated and achieves an accuracy of 91.45% with Evasion attacks, 88.55% with Poisoning attacks, 92.65% with Adversarial perturbations, and 96.33% without attacks. The security analysis of the proposed SBO Ensemble Tree Classifier based on accuracy is illustrated in Fig 11.

### 5.11 ROC analysis

**Fig 12:**
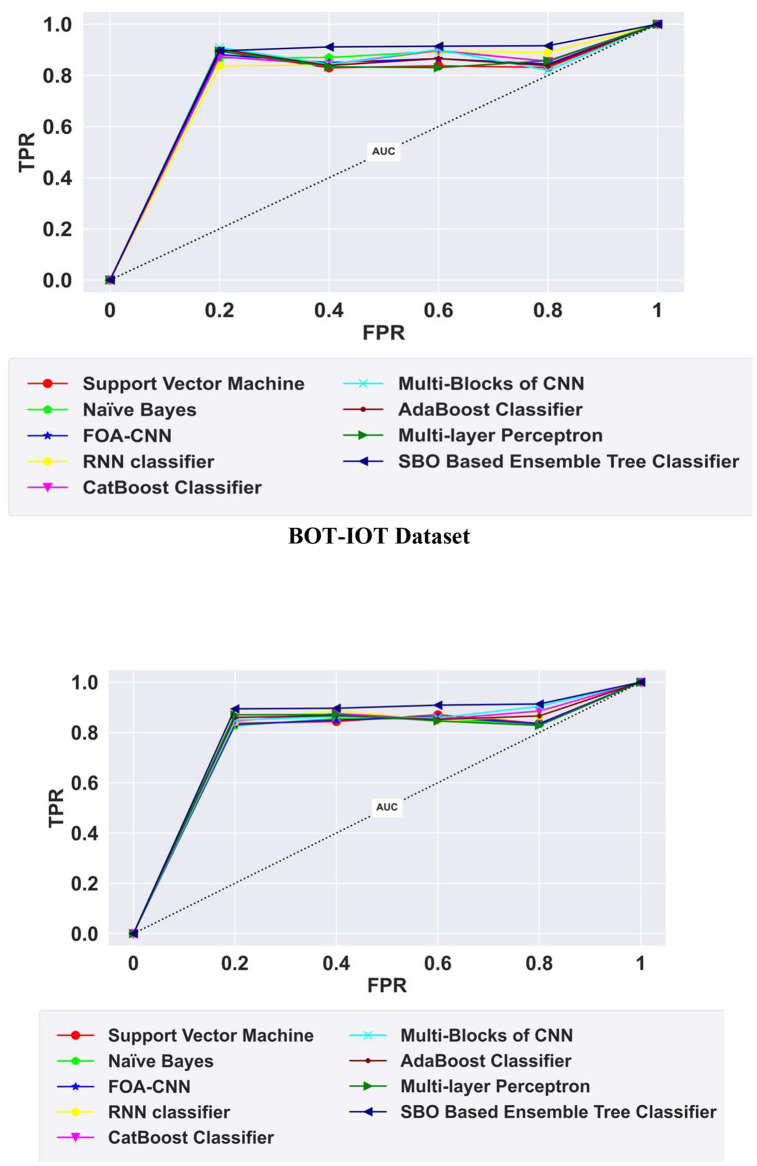
ROC Analysis.

ROC analysis of the proposed SBO-Ensemble Tree Classifier is conducted with other existing models to evaluate the performance of the model for correctly detecting intrusions in terms of True Positive Rate (TPR) and False Positive Rate (FPR). Here, TPR denotes the sensitivity of the classifier that correctly detects intrusion, and FPR denotes the proportion of incorrect intrusion detection. The proposed SBO-based ensemble Tree Classifier achieved a high sensitivity of 0.92% for the BOT-IOT Dataset, and 0.91% for the CIC-IDS-2017 Dataset, which explains the high efficiency of the classifier and outperforms the other existing models. Fig 12 illustrates the ROC analysis of the proposed SBO-based ensemble Tree Classifier.

## 6. Conclusion

In conclusion, this research introduces an all-encompassing and innovative SBO-based ensemble tree classifier for detecting intrusions in VANETs. The integration of a VANET simulation, data preprocessing, and a sophisticated tree-based ensemble classifier comprising decision trees, random forests, extra trees, and XG Boost demonstrates a systematic methodology for identifying and categorizing potential attacks within the VANET environment. The introduction of a hybrid optimization model, combining TLBO and CSO Optimization, enriches the intrusion detection process by optimizing the outputs from the ensemble classifier. This holistic framework addresses the challenges of accuracy, efficiency, and adaptability in intrusion detection, offering a robust security solution for vehicular communication systems. The research contributes significantly to the advancement of intrusion detection methodologies, inspiring further exploration and development in securing connected vehicle networks against evolving cyber threats. The novel integration of nature-inspired optimization techniques and a sophisticated ensemble classifier represents a noteworthy contribution to the field, paving the way for enhanced security measures in the dynamic landscape of VANETs. More specifically, the proposed SBO-based ensemble tree classifier achieved high performance for intrusion detection with an accuracy of 96.56%, F1-score of 96.63%, FPR of 0.97, MCC of 0.97, Precision of 96.59%, Sensitivity of 96.68%, and Specificity of 96.52% using the BOT-IOT Dataset.
